# Decreasing Antibiotic Use in a Community Neonatal Intensive Care Unit: A Quality Improvement Initiative

**DOI:** 10.1055/a-2158-8422

**Published:** 2023-09-25

**Authors:** Harjinder P. Singh, Susan Wilkinson, Shahid Kamran

**Affiliations:** 1 Division of Neonatology, Pomona Valley Hospital Medical Center, Pomona, California

**Keywords:** antibiotic stewardship program, antibiotic use rate, neonatal early-onset sepsis, neonatal intensive care unit, sepsis risk calculator

## Abstract

**Objective** In view of the excessive use of antibiotics in our neonatal intensive care unit (NICU), we launched a 5-year multidisciplinary quality improvement (QI) initiative in our NICU in 2018. We had set our aim of decreasing the antibiotic use rate (AUR) from 22 to 17%.

**Study Design** The QI initiative was conducted in our 53-bed level 3B NICU. We used the core elements of antibiotic stewardship and focused on improving gaps in knowledge by using updated standards of care and a multidisciplinary approach. Outcome measures included overall AUR in NICU. Statistical control chart (P chart) was used to plot the AUR data quarterly.

**Results** The AUR demonstrated a decline at the onset, and at the end of the initiative the AUR demonstrated a sustained decline to 13.18%, a 40% decrease from the baseline AUR of 22%. The changes that were implemented included development of evidence-based guidelines for babies less than and greater than 35 weeks, daily antibiotic stewardship rounds, sepsis risk calculator, antibiotic stop orders (48-hour stop, 36-hour soft stop, and 36-hour hard stop), and periodic reviews.

**Conclusion** Our multidisciplinary approach using all the core elements of an antibiotic stewardship program significantly decreased AUR in our NICU.

**Key Points**

Excessive use of antibiotics may cause harm to the infant's health.

Indiscriminate use of antibiotics can lead to antibiotic resistance.

Stewardship programs can significantly decrease AUR in NICUs.

The incidence of neonatal early-onset sepsis (EOS) has declined by nearly four-fold since guidelines from the Centers for Disease Control and Prevention (CDC) and the American Academy of Pediatrics (AAP) recommended group B Streptococcus antibiotics prophylaxis. 1 2 3 However, this has led to increased use of laboratory testing and antibiotics for bloodstream infections in newborn infants, many of whom show no clinical symptoms of sepsis. A negative blood culture is required to discontinue antibiotics after 48 hours. Even in cases with a negative culture result, the lack of consensus regarding antibiotic use in newborns with suspected sepsis often results in prolonged antibiotic use. 4 The fact that only 3 to 8% of those screened for EOS have culture-proven sepsis highlights the cautious nature of conventional approaches to the management of neonatal sepsis. 5 6

The prolonged use of broad-spectrum antibiotics is not without consequences. Prolonged exposure to antibiotics can disrupt the normal flora in the gut, leading to dysbiosis and selection of more aggressive and pathogenic bacteria. 7 Cotten et al showed that infants receiving more than 5 days of antibiotics for EOS had increased risk for developing necrotizing enterocolitis. 8 Indiscriminate use of antibiotics can lead to antibiotic resistance. 9 10 There is also concern for long-term consequences of early antibiotic exposure, including the development of obesity later in life. 11 12 13 Antibiotic prescribing practices vary widely among neonatal intensive care units (NICUs), from 2.4 to 97.1%. 14 15 However, despite the wide range of antibiotic prescribing practices, clinical outcomes remain similar, suggesting that antibiotics are often prescribed unnecessarily. 14 15

In the last few years, there has been an organized approach nationally to decrease antibiotic exposure to help reduce the development of antibiotic resistance. While the experience of antibiotic stewardship programs (ASPs) for adult populations have been extensively reported, 16 17 18 there have been fewer reports in the newborn population, especially those in nonacademic community NICUs. 19 20 21 Our NICU had been tracking antibiotic use rate (AUR) on a yearly basis for all antibiotic usage including EOS and late-onset sepsis. AUR of 22% for all antibiotic exposure was identified as a significant problem at the Pomona Valley Hospital Medical Center (PVHMC) NICU in 2018. This was reviewed in the context of low incidence of EOS in our NICU of 0.6/1,000 live births in the preceding years. We believe that lack of standardized antibiotic prescription guidelines as well as lack of consensus on when to stop antibiotics with negative cultures contributed to excessive use of antibiotics.

Schulman et al noted that in NICU's with ASPs, the AUR declined by 28.7% as compared with 16.2% decline in NICU's that did not have an ASP. 15 Hence, we set out to revise our institutional methodology in ways that would decrease antibiotic exposure for newborn infants with suspected sepsis in our NICU. We launched this quality improvement (QI) program based on the Institute of Health Care Improvement (IHI) model. The IHI model is a simple and effective tool used by health care organizations to promote positive change, increase quality, and reduce cost. Our aim was to decrease the overall AUR in NICU from 22% at baseline to 17% by the end of 2021.

## Materials and Methods

PVHMC NICU is a 53-bed level 3B NICU. The NICU receives referrals from Labor and Delivery Department with approximately 6,000 births annually. Neonatal and maternal transport teams bring in additional referrals from the surrounding Inland Empire region that includes the counties of Los Angeles, San Bernardino, Riverside, Mono, and Kern County. The NICU provides care to infants with gestational age of 22 weeks and higher as well as sick infants requiring surgical interventions, inhaled nitric oxide, and therapeutic hypothermia. Infants with complex heart conditions and need for extracorporeal membrane oxygenation are referred to the nearby tertiary center. Symptomatic infants with suspected EOS are admitted to the NICU. Asymptomatic babies with history of maternal chorioamnionitis are cared for in the newborn nursery and are not part of this stewardship program. The Newborn Nursery is a separate administrative unit governed directly by the Department of Pediatrics and was not included in the NICU ASP. Neonatology service is consulted when these infants become symptomatic requiring NICU care. A small number of babies may be treated for 48 hours in the newborn nursery.

## Interventions

The interventions that comprised the ASP were first implemented in 2018 ( Table 1 ). The success or failure of each intervention was critically evaluated on a quarterly basis, and interventions were modified based on the results of analysis.

**Table 1:** Change implementation timeline

Quarter	Implementation
Q3 2018	Antibiotic stewardship team
Q1 2019	Sepsis calculator Sepsis ruled out at 48 h Antibiotic stewardship rounds
Q1 2020	36-h soft stop
Q3 2020	Electronic medical record linked to the sepsis calculator and maternal information
Q4 2020	36-h hard stop for all NICU antibiotic orders

Abbreviation: NICU, neonatal intensive care unit.

We assembled a multidisciplinary NICU antibiotic stewardship team in the third quarter of 2018. The stewardship team included neonatologists, NICU medical director, NICU quality supervisor, NICU nursing staff, pharmacists, Medical Director of PVHMC infection control department, and an infection prevention nurse.

**Fig. 1:**
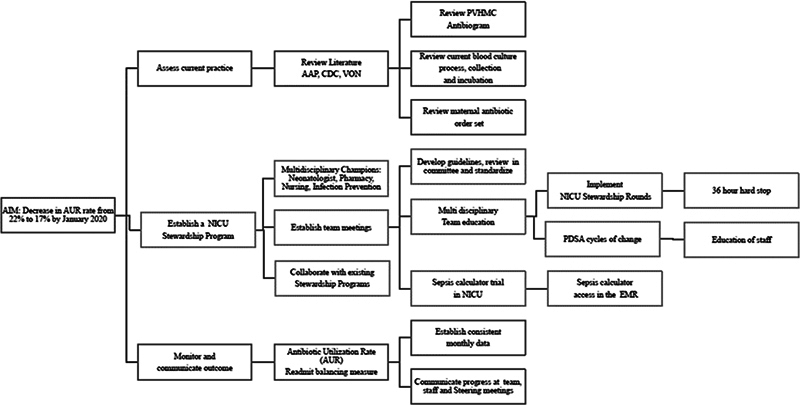
Driver diagram. AAP, American Academy of Pediatrics; CDC, Centers for Disease Control and Prevention; EMR, electronic medical record; NICU, neonatal intensive care unit; PDSA, Plan Do Study Act; PVHMC, Pomona Valley Hospital Medical Center; VON, Vermont Oxford Network.

The team met weekly for 2 months and then met monthly. The team collectively reviewed available clinical literature on EOS that included guidance from AAP. AUR data were collected quarterly and reviewed by the stewardship team. Team members also examined NICU antibiotic prescription practices in effect, the NICU specific antibiogram, and antibiotic sensitivity pattern. The team visited the affiliated tertiary center to exchange ideas with staff and learn about the practices used by the established ASP at that institution. A key driver diagram that identifies what drives or contributes to the project aim was developed ( Fig. 1 ).

In Quarter 1 (Q1) of 2019, the decision was made to update the institutional methodology for NICU antibiotic prescription.

**Fig. 2:**
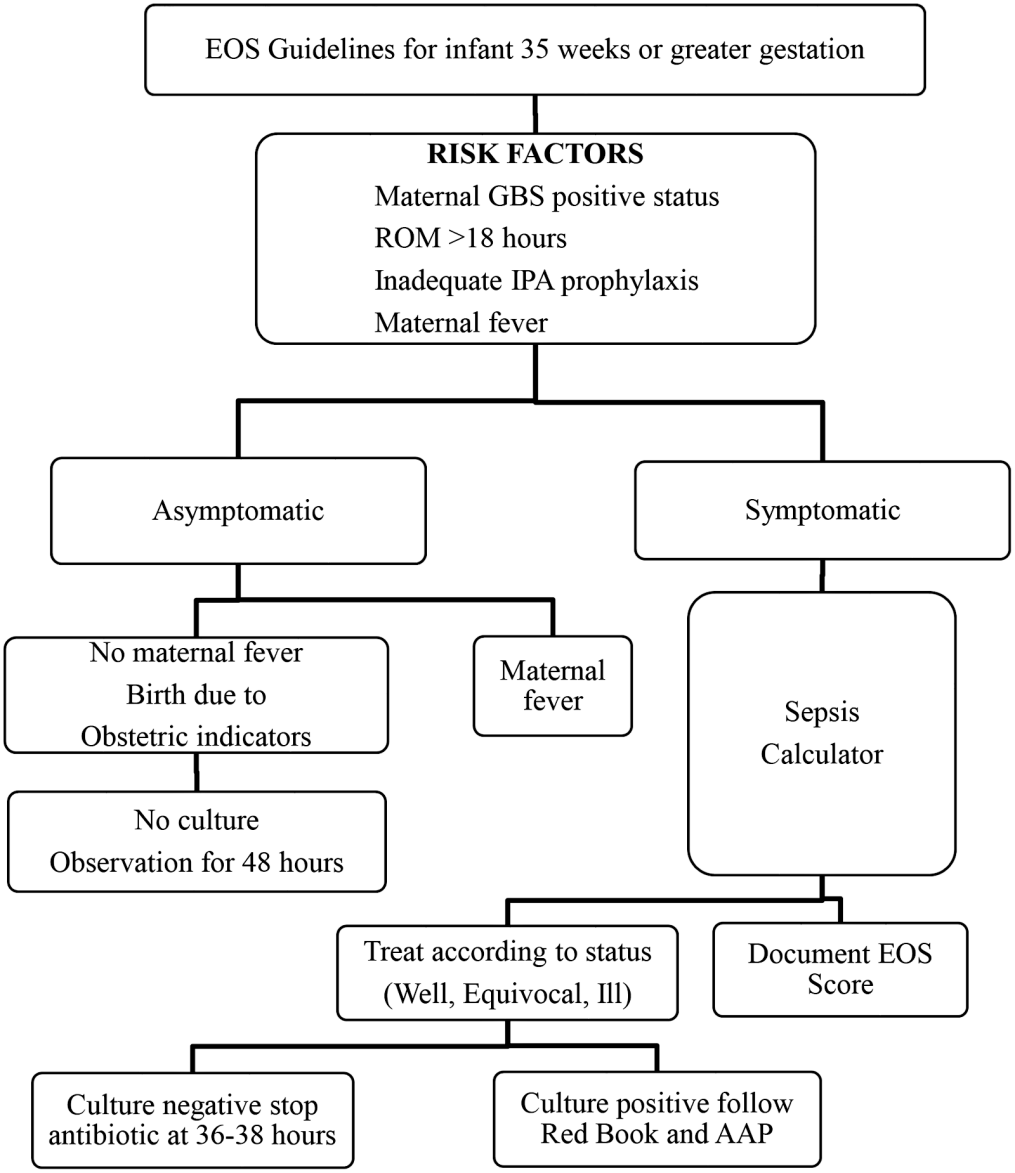
EOS guidelines for Infants 35 weeks or greater gestation. AAP, American Academy of Pediatrics; EOS, early-onset sepsis; GBS, group B streptococcus; IPA, intrapartum antibiotic; ROM, rupture of membrane.

**Fig. 3:**
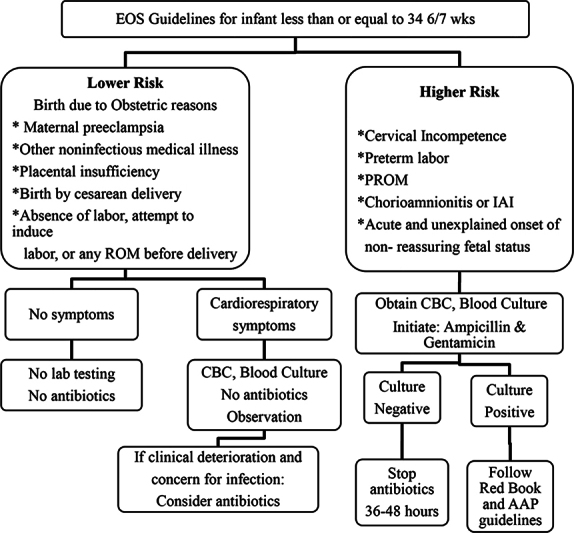
EOS guidelines for infants 34 ^6/7^ or less gestation. AAP, American Academy of Pediatrics; CBC, complete blood count; EOS, early-onset sepsis; GBS, group B streptococcus; IAI, intra-amniotic infection; PROM, premature rupture of membranes; ROM, rupture of membrane.

Review of our data led to the development of strategies for improvement that included creation of standardized guidelines for management of suspected sepsis ( Figs. 2 and 3 ), 22 periodic reviews, implementation of antibiotic stewardship rounds, use of Plan Do Study Act (PDSA) cycles, and review of any patients that required readmission due to sepsis-related illness. The guidelines for EOS included the use of sepsis risk calculator (sepsiscalculator.kaiserpermanente.org). Physician use of Kaiser sepsis risk calculator was followed on a monthly basis. Sepsis evaluation generally included a complete blood count with manual differential count and a blood culture obtained via either peripheral vein or an umbilical catheter when applicable.

Stewardship rounds initially included pharmacists rounding with the attending physicians.

The pharmacist collected pertinent clinical and laboratory data on a daily basis for any baby on antibiotics, and data were reviewed with the team of physicians. A negative blood culture at 48 hours was considered sufficient evidence for the discontinuation of antibiotic treatment. The pharmacist assumed the responsibility of checking the culture results at 48 hours and discontinuing the antibiotics. If antibiotics were continued beyond 48 hours despite negative blood culture results, physicians were instructed to document the reasoning for continued antibiotic use in the electronic medical record (EMR). The pharmacist created a stewardship document in the EMR each day to document the consensus reached at the stewardship rounds.

## 2019

### Sepsis Probability Risk Calculator

**Fig. 4:**
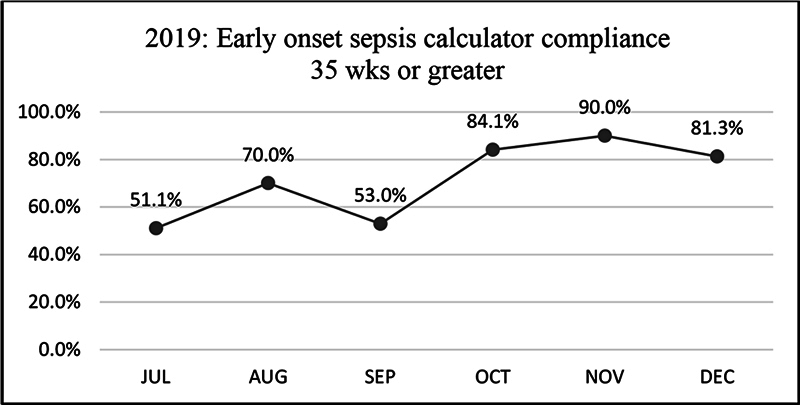
2019 early-onset sepsis calculator compliance 35 weeks or greater.

Treatment of infants 35 weeks or greater was directed by the use of the EOS calculator and documented EOS risk per 1,000 births. Chart review revealed poor physician compliance with use and documentation of the EOS risk. In Q3 2019 physicians were asked for feedback on the EOS calculator use. The physicians reported that navigating to another website outside the EMR in order to use the sepsis calculator was cumbersome and time consuming. The need for a process that provided easier access to obtain the maternal information for the calculator was noted. In the meantime, physicians were reeducated about the need to document an EOS risk on all infants greater than or equal to 35 weeks in the EMR and treat accordingly. Education took place during handoff rounds, via email communications, and during discussions at monthly NICU meetings. The Medical Director completed review of cases with missing EOS risk documentation and provided follow up. We noted increased use of the EOS calculator, from 51.1% in Q3 2019 to 81 to 90% by Q4 2019 ( Fig. 4 ). The EOS calculator web page was linked to the EMR in Q3 2020, which improved the overall process and efficiency for the providers. Compliance with the sepsis calculator was noted 90% or greater in Q4 2020.

### Antibiotic Stewardship Rounds

The antibiotic stewardship rounds were introduced in Q1 2019. These rounds were designed to have a daily group discussion between the pharmacist and the physicians rounding on a particular day. In Q4 2019, a review of the stewardship rounding process revealed that pharmacists were often rounding with individual physicians rather than the whole team. To address this inconsistency, the stewardship team established a consistent time and place for the rounding process. The stewardship rounds were incorporated into the daily physician handoff that occurred every morning. All physicians that would be working that day, as well as the physician on call the previous night, were present for the handoff between shifts. The pharmacist that would be working that day also participated in rounds. All infant cases scheduled to receive antibiotics that day were discussed. Antibiotic treatment was discontinued based on the consensus reached during these rounds. The pharmacist was responsible for daily creation of a stewardship progress note and for placing any required orders for the discontinuation of antibiotic treatment.

### Antibiotic Dosages

**Fig. 5:**
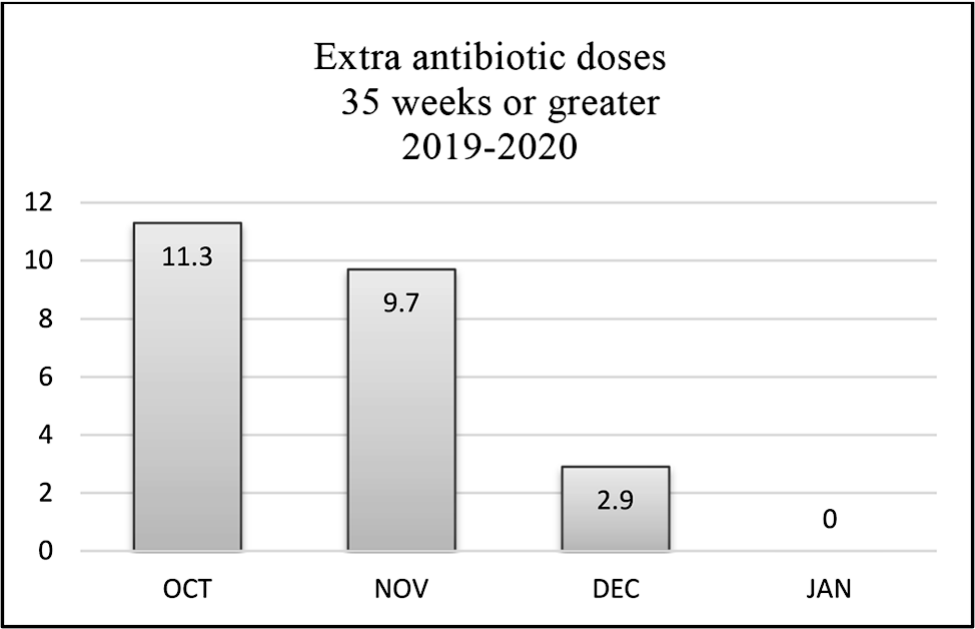
Extra antibiotic dosages 35 weeks or greater 2019 to 2020.

In Q4 2019, review of the October data revealed that 11.3% of infants with gestational age >35 weeks had received unintended extra doses of antibiotics. Review of these cases with the pharmacy manager identified poor oversight and poor coverage over the weekends as contributing factors. A core pharmacy team was developed. Weekend coverage was improved by staffing core pharmacy team members that were knowledgeable about the use of the sepsis risk calculator. Institutional protocol was updated to require stewardship rounds be increased from 5 to 7 days a week. This resulted in a progressive decrease of unintended doses administered during Q4 2019 ( Fig. 5 ).

## 2020

### 36-Hour Stop

In Q1 2020, the 48-hour antibiotic stop was transitioned to a 36-hour stop. With this modification to institutional protocol, the pharmacist was expected to place the 36-hour stop after stewardship rounds if it was ascertained that sepsis had been ruled out.

### 36-Hour Hard Stop

In Q1 2020, it was noted that the 36-hour stop was not being followed consistently. Some physicians were uncomfortable discontinuing the antibiotics at 36 hours.

To address this concern, the hospital's infection control department was asked to review all positive blood cultures in the NICU for the previous year. This review concluded that in the newborn population, all positive cultures were noted to have become positive within 36 hours. Hence, the stewardship team agreed upon a 36-hour hard stop. In Q4 2020, education to all staff about the new process took place and the 36-hour hard stop was implemented for all NICU antibiotics. In consultation with the information technology department a process was implemented for automatic discontinuation of any antibiotics prescribed in the NICU at 36 hours. If the infant required antibiotics beyond 36 hours, a new order was required to be placed in the EMR.

**Fig. 6:**
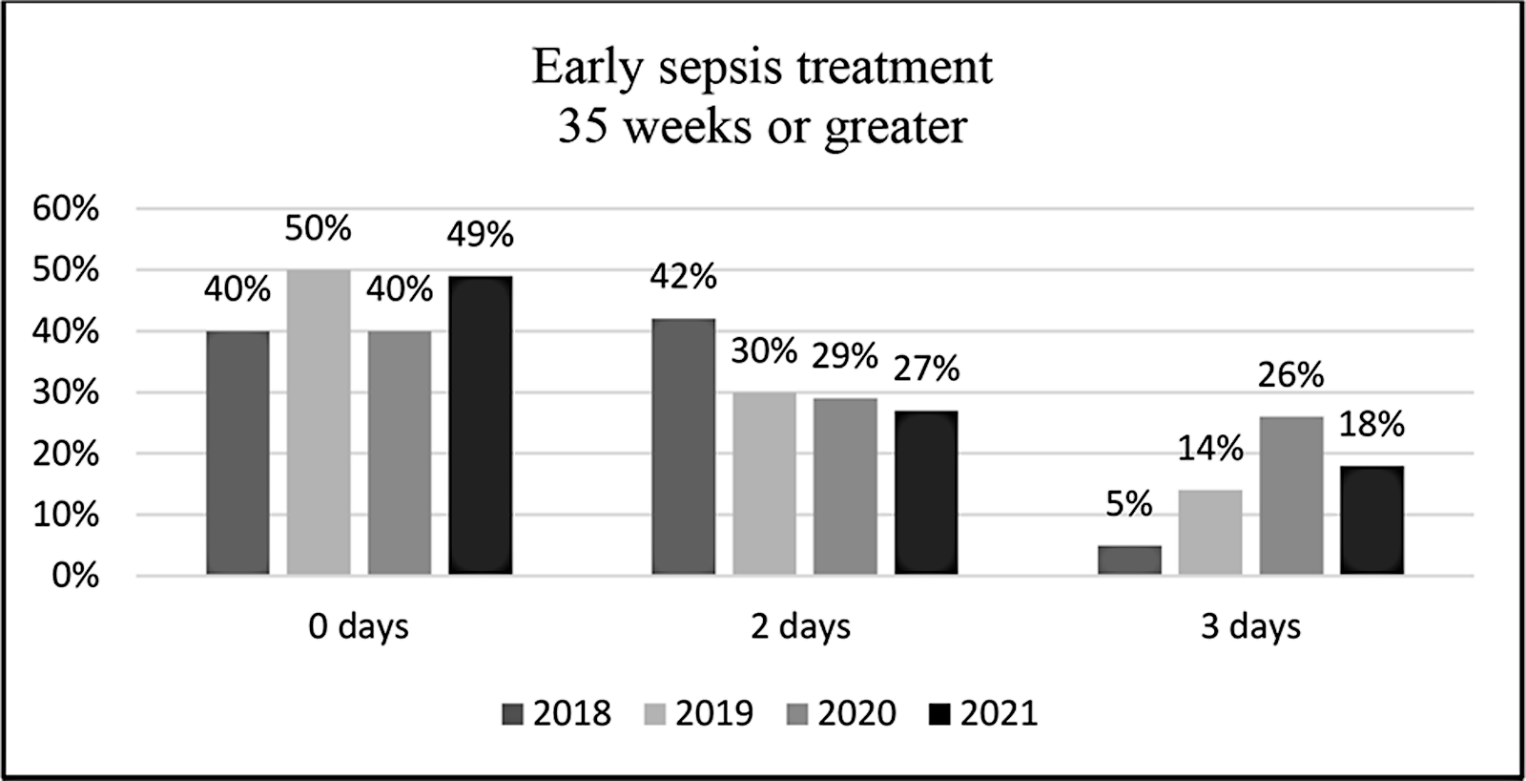
Early-onset sepsis treatment days 35 weeks or greater.

The 3-day antibiotic treatment of infants 35 weeks or greater decreased from 26% in 2020 to 18% in 2021 ( Fig. 6 ).

### External Link

To streamline the process of obtaining maternal information and the sepsis calculator use, an external link was created in the EMR. The external link directs the physician to maternal data needed for the sepsis calculator consolidated in one place. A link to the Kaiser sepsis calculator was also inserted on the same page that would open up the sepsis calculator without having to navigate out of the EMR.

### Analysis

Data were obtained from the NICU EMR system. Statistical control charts (P Chart) was used to plot the AUR quarterly. The data on sepsis-related readmissions were also obtained from the EMR.

## Ethical Considerations

The institutional review board (IRB) at PVHMC approved the study. The protocol submitted to the IRB was updated periodically to document project progress.

## Results

### Primary Measures

**Fig. 7:**
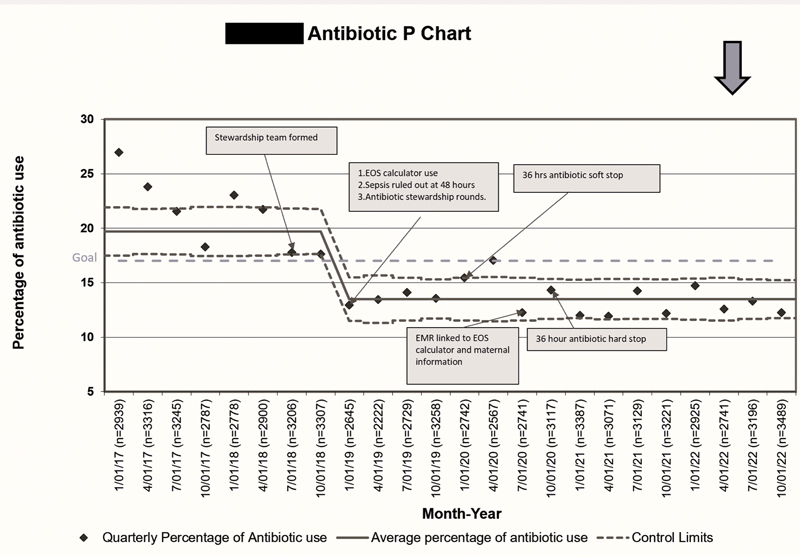
Antibiotic use rate P chart.

The primary outcome measure was AUR in the NICU. AUR is calculated as a percentage total number of NICU days of parenteral antibiotic exposure, divided by the total number of NICU days for that year. A decrease in AUR was noticed after the antibiotic stewardship program was implemented in July 2018 ( Fig. 7 ). In the fourth quarter of 2018, while the literature was being reviewed, the physicians became aware of the most recent guidelines for treating suspected sepsis. The practice of antibiotic prescription began to change as newer guidelines were being made. By the end of Q1 2019, the sepsis calculator, the 48-hour stop, and the antibiotic stewardship rounds were formally put into practice. Further decline in AUR was noticed after these interventions.

After the initial interventions of sepsis calculator use, antibiotic stewardship rounds, and 48-hour antibiotic stop, the AUR demonstrated a sustained decrease. Further interventions including the introduction of an external link to the sepsis calculator, 36-hour stop, and a 36-hour hard stop helped sustain the results ( Fig. 7 ). The decrease in AUR was sustained through the years of stewardship program from 2019 to 2022 ( Fig. 7 ). By the end of the year 2022, the decrease in AUR was sustained and low at 13.18%, reduction of approximately 40% from the baseline before beginning the stewardship program.

During the years of 2018 to 2022, our NICU admission rate ranged between 675 and 762 patients per year. Our inborn admission rate ranged between 11 and 13% of live births. The NICU mortality rate ranged between 9 and 12 per 1,000 admissions. The number of patients less than 36 weeks ranged between 285 and 386 patients per year.

The algorithm in Fig. 3 was developed as a guideline for treatment of infants 34 weeks or less. The goal was to standardize and decrease the length of treatment with antibiotics.

Although we did not measure compliance with this guideline, we observed a decrease in the length of antibiotic treatment. In infants 34 weeks or less gestation, the antibiotic treatment showed improvement over the years of ASP. Infants with no treatment did not show any significant change (36% in 2019 vs. 33% in 2022). Infants with 2-day treatment showed improvement from 38% in 2019 to 58% in 2022. We believe that some of these infants were originally being treated for 3 days or more. The proportion of infants treated for 3 days demonstrated much desired change from 21% in 2019 down to 5% in 2022. Infants receiving 7 days or longer treatment remained nearly the same 3 versus 2% for 7-day treatment and unchanged at 2% for treatment greater than 7 days. We believe that the lesser proportion of babies treated for 3 days resulted in decreased length of treatment overall in this group of infants.

### Secondary Measures

Sepsis-related readmissions to the NICU were utilized as a balancing measure. There were no sepsis-related cases admitted during the study period ( Table 2 ). We were limited to readmission data at PVHMC. However, since the majority of these infants belong to PVHMC health service area, we are confident that they would be readmitted to our facility.

**Table 2:** Readmissions

Year	Total admission	Sepsis readmission	Other readmission
2018	732	0	2 Respiratory distress
2019	689	0	2 CLD, 2 hyperbilirubinemia
2020	675	0	1 Emesis, 2 hyperbilirubinemia
2021	762	0	1 desaturation 1 hyperbilirubinemia

Abbreviation: CLD, chronic lung disease.

As an additional finding, we reviewed infants with gestational age > 35 weeks treated with antibiotics for more than 2 days. The 3-day antibiotic treatment of infants 35 weeks or greater decreased from 26% in 2020 to 18% in 2021 ( Fig. 6 ).

## Discussion

Decreasing antibiotic use in the NICU is a challenging task. The contributing factors include absence of specific guidelines to initiate antibiotics in infants with suspected sepsis and the lack of consensus on when to discontinue antibiotics in infants with unproven sepsis.

Antibiotic stewardship interventions so far have been mostly based on established treatment guidelines, 23 24 25 automatic antibiotic stop date, 26 27 28 prospective audit, 19 and use of sepsis risk calculator. 29 30 A recent meta-analysis report showed that use of the neonatal EOS calculator is associated with a substantial reduction in the use of empirical antibiotics for suspected EOS. 31 Recently, more comprehensive stewardship reports have been published. 16 17 We devised our ASP based on the CDC guidance for hospital ASPs in 2019. We incorporated all the core elements recommended by the CDC that included leadership commitment, reporting, tracking, active oversight, pharmacy expertise, accountability, and education. Our stewardship program included daily antibiotic stewardship rounds, staff education, data review, and an overall periodic review of the program for compliance. Our approach to management of suspected sepsis was based on the guidelines recommended by the AAP Committee on the Fetus and Newborn 2018. We were able to gradually foster a comprehensive and multidisciplinary approach to the use of antibiotics by initiating the ASP, which eventually became a standard of practice in our NICU. Team compliance was achieved via education at daily stewardship rounds, email notification, discussion during daily work rounds, and medical director review of noncompliant cases. The role of pharmacy department was crucial in implementing the interventions. Hence, a core team of pharmacists was assembled at the direction of the hospital's pharmacy department. This core team was actively involved in the development and implementation of initial measures and interventions. The rest of the pharmacy team members that would rotate through the NICU were also periodically educated and updated on the procedures of the program. The pharmacy department made certain adjustments in their scheduling in order to enable the core pharmacy team members to rotate through the NICU. Hence, no additional funding was needed on behalf of the pharmacy. Originally adopting an aim statement, a key driver diagram, testing changes with PDSA cycles, and measuring data, we observed a gradual and sustained improvement in the AUR for 3 years, suggesting an actual change. In Q2 2020, the AUR was noted to increase up to the mean. Upon review, we found multiple culture positive cases for that quarter with complicated bowel diagnoses, congenital syphilis, pneumonia, meningitis, and cellulitis that contributed to longer antibiotic treatment. The most recent AUR of 13.18% in the year 2022 surpassed our initial aim of 17%. We believe that the use of standardized guidelines for antibiotic management and institution of daily antibiotic stewardship rounds helped us significantly in tracking the progress as well as identification of problems on a daily basis. Variations in the use of antibiotic management was discussed at stewardship rounds, as well as reviewed by the medical director of the NICU.

In addition, a summary of review was presented monthly at the NICU steering committee meetings. Implementation of various interventions including use of a sepsis risk calculator, daily antibiotic stewardship rounds, and antibiotic stop orders and testing the change resulted in a sustained decrease in AUR.

Antibiotic stewardship is not new, but our program employed a multidisciplinary approach, enlisting the expertise of all stakeholders. We employed established QI processes and methods, including the IHI model and PDSA cycles of change. A 40% reduction in AUR in our NICU prevented unnecessary use of antibiotics in many infants. By achieving sustained improvements in AUR beyond our stated goal, we were able to change the culture of antibiotic use in our NICU and implement improved and standardized practices of antibiotic management.

## Limitations

In the beginning, the challenges in accessing the maternal data as well as the sepsis calculator resulted in poor compliance with the use of sepsis calculator. Our stewardship program was implemented only in the NICU, and hence, the antibiotic use in the newborn nursery is not addressed in this program. Other NICUs and our larger group were not part of this program and those other units implement their own ASP based on their local situation and practices.

## Conclusion

Our approach to decreased antibiotic use in the NICU was devised after thorough evaluation of our practices and application of evidence-based standards of care. A comprehensive and multidisciplinary QI-based approach to antibiotic use in our NICU significantly reduced the AUR. We believe that all NICUs should devise their own ASP based on the existing practices and available resources using established QI processes.
